# Prevalence and risk factors of *Schistosoma mansoni* infection among preschool-aged children from Panamasso village, Burkina Faso

**DOI:** 10.1186/s13071-021-04692-8

**Published:** 2021-04-01

**Authors:** Mamoudou Cisse, Ibrahim Sangare, Arthur D. Djibougou, Marc C. Tahita, Souleymane Gnissi, Jonathan K. W. Bassinga, Salifou Konda, Abdoulaye H. Diallo

**Affiliations:** 1 Centre MURAZ, Institut National de Santé Publique, Bobo-Dioulasso, Burkina Faso; 2 Institut Supérieur des sciences de la Santé, Université Nazi BONI, Bobo-Dioulasso, Burkina Faso; 3 Unité de Recherche Clinique de Nanoro, Institut de Recherche en Sciences de la Santé, Nanoro, Burkina Faso; 4 Unité de Formation et de Recherche en Sciences De la Santé, Université Joseph KI-ZERBO, Ouagadougou, Burkina Faso

**Keywords:** *Schistosoma mansoni*, Prevalence, Risk factors, Preschool-aged children, Burkina Faso

## Abstract

**Background:**

Schistosomiasis remains a major public health concern in sub-Saharan Africa. Although schistosomiasis is well documented in school-aged children in Burkina Faso, prevalence data among preschool-aged children (PSAC) are limited and outdated, and its risk factors in this group remain poorly documented. The main objective of this study was to assess the prevalence and risk factors associated with *Schistosoma (S.) mansoni* infection among PSAC from Panamasso village, western Burkina Faso.

**Methodology:**

A cross-sectional study was carried out among 228 children under 6 years old from Panamasso village. Sociodemographic and water contact data were collected using a structured questionnaire. Kato-Katz and formol-ether concentration techniques were used to detect *S. mansoni* eggs in stool samples. Urine samples were subjected to a point-of-care circulating cathodic antigen (POC-CCA) cassette test and a centrifugation method to check for both *S. mansoni* and *S. haematobium* infection, respectively. Potential risk factors for *S. mansoni* infection were explored using multivariable logistic regression.

**Results:**

The mean age of children was 40.2 ± 15.0 months. The prevalence of *S. mansoni* infection as determined by Kato-Katz, formol-ether concentration and POC-CCA was 42.1%, 39.5% and 80.7%, respectively. Based on the combined results of the three methods, the overall prevalence of *S. mansoni* infection was 81.1%. No case of *S. haematobium* infection was found. The geometric mean intensity of *S. mansoni* infection was 107.2 eggs per gram of feces with 54.2%, 33.3% and 12.5% of the children having light, moderate and heavy infections, respectively. Girls (AOR = 2.9, 95% CI 1.3–6.1), a household located within 500 m from the pond (AOR = 3.0, 95% CI 1.0–8.6) or between 500 and 1000 m from the pond (AOR = 3.0, 95% CI 1.2–7.2), and the child’s history of going to the pond (AOR = 5.0, 95% CI 1.7–14.3) were the variables significantly associated with *S. mansoni* infection.

**Conclusion:**

*S. mansoni* was the sole species infecting a high proportion of PSAC in the study area. A mass drug administration program with praziquantel is therefore urgently required for those below 6 years old. Other control strategies should include increased community-awareness and provision of safe water.
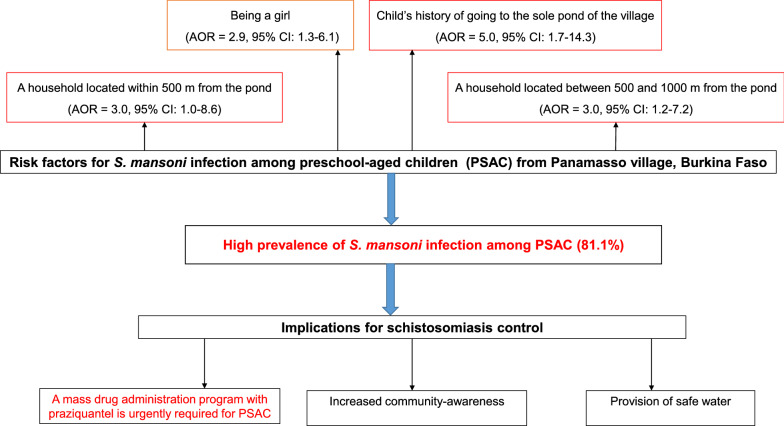

**Supplementary Information:**

The online version contains supplementary material available at 10.1186/s13071-021-04692-8.

## Background

Schistosomiasis is a major public health concern in sub-Saharan Africa (SSA) with around a third of the annual 192 million cases of schistosomiasis caused by *S. mansoni*, the causative agent of intestinal schistosomiasis [1]. About 4.4 million people infected with *S. mansoni* suffer from bloody diarrhea, while 8.5 million people have hepatomegaly associated with periportal fibrosis, portal hypertension and hematemesis [2, 3]. In addition, it is estimated that each year 130,000 deaths occur due to complications of the disease [4, 5]. In school-aged children (SAC) (i.e. 6–15 years), chronic infections with *S. mansoni* cause anemia, growth retardation and cognitive impairment [5]. The main morbidities due to *S. mansoni* infection in preschool-aged children (PSAC), i.e. under 6 years, are anemia, hepatosplenomegaly and hepatic fibrosis with a higher risk in PSAC of the 37–60-month age group [6].

PSAC have been considered a low risk group for *S. mansoni* infection [7, 8] and therefore have so far been excluded from various schistosomiasis control programs in Africa, resulting in a health inequity in affected people [9]. Although several studies have reported prevalences of *S. mansoni* infection ranging from 0.9 to 86% in PSAC [6, 10–19], the risk factors associated with this parasite infection remain poorly studied [11, 13, 15, 19, 20].

In Burkina Faso, *S. mansoni* infection was endemic in the western and southern areas of the country with a focal distribution before the implementation of the National Schistosomiasis Control Program (NSCP) in 2004 [21]. The program mainly focused on mass drug administration (MDA) with praziquantel in SAC with a coverage rate > 90% [22]. In 2010, the prevalence of *S. mansoni* infection among SAC ranged from 3.3 to 39.1% with the highest prevalence (39.1%) recorded in Panamasso village [23]. In this village, the prevalence in SAC was 26.2% after many years of MDA with praziquantel in 2013 [24].

The abundant literature on schistosomiasis infections among SAC in Burkina Faso contrasts with the scarce and outdated data on the PSAC group [25]. In addition, risk factors associated with *S. mansoni* infection are so far unknown. This study sought to fill this knowledge gap with the goal of including these children into the MDA program with praziquantel.

## Methods

### Study design, area and population

A community-based cross-sectional study was carried out in PSAC living in Panamasso from February to March 2020. Panamasso is a village located in Dafra District 30 km from Bobo-Dioulasso, the second largest city of Burkina Faso, at 11°23′0" N, 4°12′0" W, at an altitude of 337 m above sea level. This locality is located on the edge of one of the tributaries of the Mouhoun River sheltering a pond, thus providing an environment suitable for growing of intermediate snail hosts of *S. mansoni*, i.e. *Biomphalaria pfeifferi* [21]. The climate is tropical, characterized by two seasons (a rainy season between May and October followed by a dry season from November to April) and temperatures typically ranging from 21 to 33 °C with a mean annual rainfall of 1071 mm. The vegetation is mainly dominated by wooded savanna and sparse forests [26]. Panamasso had 600 households with 4403 inhabitants in 2019 belonging to three main ethnicities: Bobo, Fulani and Mossi. People are mainly engaged in subsistence agriculture (cassava, maize, gardening). Gardening is the most important agricultural activity, leading to a high frequency of contact with the pond. The pond is the main source of water for domestic activities (drinking, cooking, washing, bathing and recreation) despite well-functioning water supplies, and there is no drainage system in this village. In 2013 and in 2016, Panamasso was the only sentinel site of the NSCP to record high prevalence of *S. mansoni* infection in SAC with respective values of 39.1% and 26.2% [23, 24]. PSAC who were under the age of 6 years old were the source population. PSAC who had been living in Panamasso for at least 6 months before the study began, whose parents/guardians provided written informed consent and who had no history of having been treated with praziquantel and anti-helminthic drugs in the past 6 months were included. Children who did not provide any stool sample during the survey were not included.

### Sample size and sampling technique

Sample size was calculated upon a *S. mansoni* infection prevalence of 26.2% previously reported in SAC from Panamasso [24], a relative precision of 8%, for alpha = 0.05, and using a design effect of two. It was estimated to 230 participants accounting for 10% parent refusal. A multiple-stage sampling procedure was used to randomly select 300 households from a list of the total households of the study village, and then all eligible PSAC were included until the sample size was reached.

### Field data collection

To facilitate adhesion of community members to the study, a meeting with keys stakeholders in the local community was organized by the research team before the data collection to explain the purpose and the procedures of the study. Two weeks later, an individual structured questionnaire was administered to the selected PSAC’s parents or guardians after having obtained their informed consent. Thereafter, sociodemographic data of the PSAC and their parents’ sociodemographic data and level of knowledge about schistosomiasis as well as water contact data were collected. Water contact was assessed by inquiring about the child’s history of going to the pond, habit of swimming in the pond, frequency of swimming in the pond per week (i.e. the reported number of days in the week that the child had been to the pond) and period of swimming in the pond.

### Sample collection and laboratory procedures

Single stool and urine samples were collected in separate clean containers from each PSAC between 9 a.m. and 1 p.m. and stored at ambient temperature. Samples were thereafter transported to the Laboratory of Parasitology of Centre MURAZ within 1–4 h post-collection and processed.

#### Kato-Katz technique

One Kato-Katz thick smear was prepared from each stool sample using a template holding 41.7 mg of stool sample [27]. The slides were left for 24 h to clear for easy visualization of *S. mansoni* eggs before being examined. Infection intensity of *S. mansoni* was estimated by multiplying the total number of eggs counted by 24, which was given as the eggs per gram (epg) of feces and classified as light, moderate and heavy per the threshold set by the World Health Organization [28]. Two independent laboratory technicians examined each slide, and any resulting discrepancies were resolved by the senior parasitologist. As a quality control check, 20% of all positive and negative slides were randomly chosen and re-read by the senior parasitologist to confirm positive or negative results.

#### Formol ether concentration technique (FEC)

After diluting a small amount of stool (about 2 g) in 7 ml of 10% salt formalin solution (mixture of 100 ml pure formalin, 900 ml distilled water and 8.5 g sodium), the mixture was sieved in a centrifuge tube to remove large debris, and 3 ml of ether was added to it. The mixture was stirred vigorously for 30 s and centrifuged at 2000 rpm for 2 min. Then, the supernatant was discarded and the pellet was plated, using a pipette, between a slide and coverslip with lugol and read under a light microscope using 10× and 40× objectives [29].

#### Qualitative examination for *S. mansoni* circulating cathodic antigens (CCA)

All urine samples were tested for *S. mansoni* CCA using a commercially available point-of-care (POC-CCA) cassette test (batch numbers 191031120, ICT INTERNATIONAL, Noordhoek, South Africa) performed at room temperature according to the manufacturer’s instructions on the day of the sample collection. Briefly, 2 drops of urine were added to the well of the testing cassette and allowed to absorb. The test results were read visually 20 min later. In cases when the control bands did not develop, the test was considered invalid. Invalid tests were repeated using the same urine sample. Valid tests were scored as negative or positive. Trace (weak band) was also considered positive [30]. All the tests were read independently by two laboratory technicians, and in case of discordant results, the senior parasitologist examined it until an agreement was reached.

#### Urine centrifugation method

Briefly, 10 ml urine was centrifuged at 5000 rpm for 5 min. Then, the supernatant was discarded and the pellet was plated, using a pipette, between a slide and coverslip with lugol and read under a light microscope using 10× and 40× objectives. This method was used to check for *S. haematobium* infection.

### Ethical considerations

This study was approved by the institutional ethical committee from Centre MURAZ (ref. 2019-66/MS/SG/INSP/DG/CEI). Following an explanation of the purpose, benefits and possible risks of the study, written consent was obtained from PSAC’s parents/guardians. Children who were positive for *S. mansoni* were treated free of charge with praziquantel. All the information obtained from the PSAC and their parents was treated as private and confidential, and the records were stored in a locked cabinet.

### Data analysis

Data were double entered using Microsoft Excel 2013, then cleaned and analyzed with Stata 12 software (Stata Corp., College Station, TX, USA).

*Schistosoma mansoni* infection was defined as any positive test result irrespective of the laboratory methods used. Pearson's chi-square test or Fisher's exact test was used to compare proportions between groups. Geometric means of intensity infection (GMI) and their 95% confidence intervals (95% CI) were estimated. Log-transformed mean eggs counts were compared between groups using parametric tests (Student or ANOVA) or nonparametric tests (Wilcoxon or Kruskal-Wallis), where appropriate. The Bonferroni test was used when the ANOVA test was statistically significant.

Potential risk factors for *S. mansoni* infection were screened through univariable logistic regressions when *P* < 0.2. A multivariable logistic regression model was built using a stepwise backward method by including all independent variables of the univariable analysis with *P* < 0.2 in the model. The results were presented with odds ratios (ORs) and their 95% CI. Statistical significance was set for *P* < 0.05.

## Results

### Sociodemographic characteristics of study population

A total of 228 out of 230 PSAC were included into this study. Indeed, one of the two excluded PSAC was unable to provide a stool sample while the other one was 72 months old. Of these PSAC, 118 (51.7%) were females and 110 (48.3%) males. The mean age of the PSAC was 40.2 ± 15.0 months. The majority of PSAC’s parents were illiterate (87.7%), had no knowledge about means of transmission of *S. mansoni* infection (96.5%) and did not know means of prevention of *S. mansoni* infection (98.3%) (Table 1).

**Table 1:** Sociodemographic data of study participants

Variables	Category	Frequency	(%)
Sex	Boys	110	48.3
	Girls	118	51.7
Age (in months)	12–23	44	19.3
	24–35	35	15.4
	36–47	65	28.5
	48–59	58	25.4
	60–71	26	11.4
Mothers’ educational status	No formal schooling	200	87.7
	Formal schooling	28	12.3
Mothers’ occupation	Housewife	216	94.7
	Self-employed	11	4.8
	Employed	1	0.5
Mothers’ knowledge about means of transmission	No	220	96.5
	Yes	8	3.5
Mothers’ knowledge about means of prevention	No	224	98.3
	Yes	4	1.7

### Prevalence of *S. mansoni* and *S. haematobium* infection

Based on the Kato-Katz technique, 42.1% (96/228) PSAC were found to be infected with *S. mansoni* whereas 90 out of 228 (39.5%) were found to be infected by the FEC technique. Prevalence of *S. mansoni* infection based on the POC-CCA test was 80.7% (180/223) (Table 2). The youngest child found to be infected with *S. mansoni* based on each of the three methods was 15 months old.

**Table 2:** Prevalence of *S. mansoni* infection per used laboratory method

Techniques	Frequency	Number of infected	Prevalence (%)
Kato-Katz	228	96	42.1
FEC	228	90	39.5
POC-CCA	223^a^	180	80.7
Combined three methods	228	185	81.1

^a^Five PSAC did not provide a urine sample

The overall prevalence of *S. mansoni* infection based on the combined results of the three methods was 81.1% (185/228). The overall prevalence of *S. mansoni* infection in girls (87.3%) was significantly higher compared to that of boys (74.6%, *P* = 0.014). The overall prevalence of *S. mansoni* infection was higher in the 36–47-month age group than in children in other age groups. However, the difference was not statistically significant (*P* = 0.767).

No case of *S. haematobium* was found.

### Intensity of *S. mansoni* infection

The overall GMI for the study participants was 107.2 epg (95% CI 84.0–136.8). The GMI did not differ between girls (129.2 epg [95% CI 90.94–183.6]) and boys (85.2 epg [95% CI 61.1–118.8]) (*P* = 0.092). The GMI increased with age, and PSAC in the 60–71-month age group had the significantly highest GMI (F = 2.73, *P* = 0.034) (Table 3). Using Bonferroni’s test, the GMI differed significantly between children in the 60–71-month and 12–23-month age groups (*P* = 0.02).

**Table 3:** Relationship between the GMI and the sociodemographic and water contact data

Variables	Number of infected	GMI	95% CI	*P* value
Sex				0.092^b^
Boys	43	85.2	61.1–118.8	
Girls	53	129.2	90.9–183.6	
Age (in months)				0.034^a^
12–23	16	63.9	37.5–108.9	
24–35	14	85.8	51.2–143.6	
36–47	34	116.0	69.9–192.5	
48–59	23	104.1	66.9–162.0	
60–71	9	305.7	172.4–542.2	
Child’s shoe wearing habit				0.356^b^
No at all	8	161.5	57.6–452.7	
Always	88	103.3	80.1–133.2	
Distance of home from the pond				0.996^d^
< 500 m	22	118.3	58.0–241.5	
500–1000 m	64	105.7	80.7–138.4	
> 1000 m	10	94.8	45.6–197.1	
Go to the pond				0.866^b^
No	3	124.6	61.9–250.9	
Yes	93	106.7	83.0–137.2	
Child’s swimming habit in the pond				0.701^c^
No	13	87.7	56.8–135.3	
Yes	83	110.7	84.0–145.8	
Child’s swimming frequency per week				0.590^a^
Never	13	87.7	56.8–135.3	
Moderate (1–3)	16	139.1	69.0–280.3	
High (4–7)	67	104.8	77.2–142.3	
Child’s swimming period in a day				0.920^d^
Never	13	87.7	56.8–135.3	
7 a.m.–10 a.m	31	118.6	69.2–203.4	
11 a.m.–2 p.m	27	114.7	77.6–169.3	
3 p.m.–6 p.m	25	97.8	57.4–166.6	

^a^ANOVA test

^b^Student’s test

^c^Wilcoxon test

^d^Kruskal-Wallis test

**Fig. 1:**
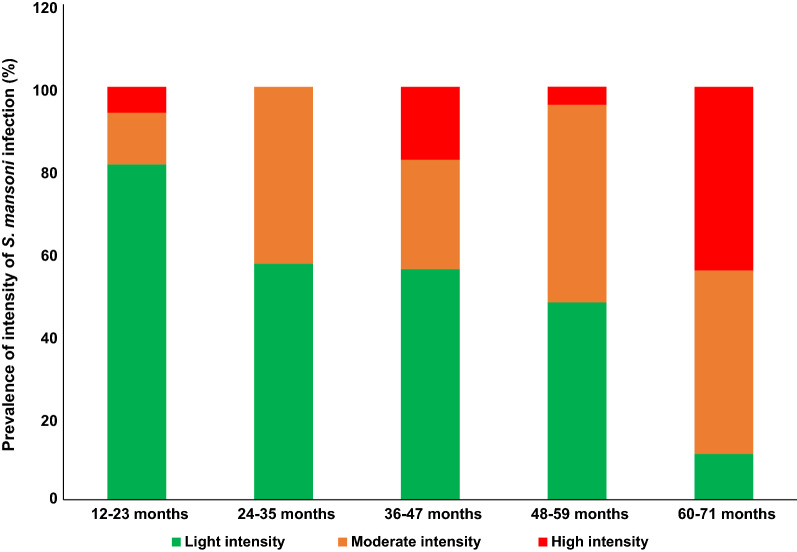
Prevalence of intensity of *S. mansoni* infection stratified by age

Among the 96 children with *S. mansoni* infection based on the Kato-Katz technique, 54.2%, 33.3% and 12.5% had light, moderate and heavy infections, respectively. In addition, the prevalence of heavy infection was significantly higher in children in the 60–71-month age group than in children in other age groups (*P* = 0.007) (Fig. 1). In contrast, the prevalence of light infection was significantly higher in children in the 12–23-month age group than in the other age groups (*P* = 0.007).

### Other intestinal parasites encountered

Based on the Kato-Katz technique, 2.6% (6/228) of the PSAC were found to be infected with intestinal helminths, and *Enterobius vermicularis* (1.8%) was the predominant species. No case of protozoa infection was found (Additional file 1: Table S1).

The FEC technique showed that 40.4% (92/228) of the PSAC examined harbored cysts of protozoa including 77 cases of single and 15 cases of double parasitic infection. *Entamoeba coli* was the most common species (21.0%), followed by *Giardia intestinalis* (11.8%) and *Entamoeba histolytica minuta* (10.0%). Helminths were found in 3.1% (7/228) of PSAC, and *Hymenolepis nana* was the predominant parasite (1.3%) (Additional file 1: Table S1).

### Factors associated with *S. mansoni* infection

In univariable logistic regression analysis, girls, low distance of home from the pond, child’s history of going to the pond, child’s swimming habit in the pond, frequency swimming in the pond per week and period of swimming in the pond were significantly associated with *S. mansoni* infection (Table 4). In multivariable logistic regression (Table 4), girls (AOR = 2.9, 95% CI 1.3–6.1), a household located within 500 m from the pond (AOR = 3.0, 95% CI 1.0–8.6), or between 500 and 1000 m from the pond (AOR = 3.0, 95% CI 1.2–7.2), and child’s history of going to the pond (AOR = 5.0, 95% CI 1.7–14.3) remained the variables significantly associated with *S. mansoni* infection.

**Table 4:** Factors associated with prevalence of *S. mansoni* infection

Variables	*S. mansoni* infection	COR (95% CI)	AOR (95% CI)	*P value*
Sex				
Boys	74.6 (82/110)	1		
Girls	87.3 (103/118)	2.3 (1.2–4.7)	2.9 (1.3–6.1)	0.007
Age (in months)				
12–23	77.3 (34/44)	1		
24–35	80.0 (28/35)	1.2 (0.4–3.5)		
36–47	86.2 (56/65)	1.8 (0.7–5.0)		
48–59	81.0 (47/58)	1.3 (0.5–3.3)		
60–71	76.9 (20/26)	1.0 (0.3–3.1)		
Child’s shoe wearing habit				
Always	80.0 (16/20)	1		
No at all	81.3 (169/208)	1.1 (0.3–3.4)		
Distance of home from the pond				
> 1000 m	61.2 (30/49)	1		
500–1000 m	86.9 (113/130)	4.2 (2.0–9.1)	3.0 (1.2–7.2)	0.014
< 500 m	85.7 (42/49)	3.8 (1.4–10.2)	3.0 (1.0–8.6)	0.047
Go to the pond				
No	42.9 (9/21)	1		
Yes	85.0 (176/207)	7.6 (2.9–19.5)	5.0 (1.7–14.3)	0.003
Child’s swimming habit in the pond				
No	64.3 (27/42)	1		
Yes	85.0 (158/186)	3.1 (1.5–6.6)		
Child’s swimming frequency per week				
Never	64.3 (27/42)	1		
Moderate (1–3)	85.3 (29/34)	3.2 (1.0–10.1)		
High (4–7)	84.9 (129/152)	3.1 (1.4–6.7)		
Child’s swimming period in a day				
Never	64.3 (27/42)	1		
7 a.m.–10 a.m	87.0 (67/77)	3.7 (1.5–9.3)		
11 a.m.–2 p.m	81.5 (44/54)	2.4 (1.0–6.2)		
3 p.m.–6 p.m	85.5 (47/55)	3.3 (1.2–8.7)		
Mothers’ educational status				
Formal schooling	75.0 (21/28)	1		
No formal schooling	82.0 (164/200)	1.5 (0.6–3.8)		

## Discussion

To the best of our knowledge, this is the first study in Burkina Faso that has attempted to assess the prevalence and intensity of schistosomiasis among PSAC and to address the issue of risk factors.

The prevalence of *S. mansoni* infection based on the Kato-Katz technique in this study (42.1%) was moderate and lower than those reported in Niger (43.8%) [18], Tanzania (44, 4%) [19] and Uganda (74.9%) [6]. However, it was higher than those found in Burkina Faso (36.3%) [25] as well as in other countries in SSA ranging from 0.9 to 39.3% [11–17]. The differences in prevalence among these studies could be attributed to the types of water bodies, water contact practices, study design, time of survey, and environmental and socio-economic factors [13, 15, 17, 31]. In addition, the actual prevalence of *S. mansoni* infection in our study could be considerably higher if the Kato-Katz technique had been performed from several stool samples provided by the same study participant [6, 17].

The overall prevalence of *S. mansoni* infection observed in this study was similar to that reported in Tanzania (82.1%) [19] but lower than that reported in Uganda (89.4%) [32]. Altogether, these findings suggest that the use of only one technique including the Kato-Katz technique in PSAC may result into underestimation of the prevalence of *S. mansoni* infection. Indeed, the POC-CCA test was up to twofold more accurate than the Kato-Katz in this study, and this corroborates findings of previous studies done in other areas of moderate prevalence [17, 33]. Therefore, we recommend using the POC-CCA test alongside the Kato-Katz to accurately estimate the true prevalence of schistosomiasis in PSAC [12].

The overall GM in the present study was of moderate intensity and was lower than those reported elsewhere ranging from 111 to 5721 epg [6, 19, 20, 32]. This difference could be due to the frequency of water contact and density of infected intermediate snail hosts in the water bodies, especially during the hot hours of the day [31]. The higher GMI observed in older PSAC corroborates findings of previous studies [19, 20, 31]. Older PSAC are known to have frequent water contact [34] [13, 19] and to spend more time in infested water compared to younger children [20], thus increasing the exposure time for *S. mansoni* infection. The build-up of schistosome loads with age due the long life span of *S. mansoni* worms (approximately 10 years) could be another explanation for this higher GMI in the oldest age group [35]. In addition, the high proportion of light infection has been previously reported [6, 13, 17, 19, 20, 32] and could be due to a shorter time of exposure to infection and thus reduced worm burden and less egg excretion in PSAC [20]. It could be also attributed to the positive impact of prior MDA with praziquantel in SAC on schistosomiasis morbidity in the study area as reported elsewhere [18]. Nevertheless, heavy *S. mansoni* infection was observed even in younger PSAC (i.e. those in the 12–23-month age group) with a prevalence of 6.3%. Our findings corroborate those of Ruganuza et al. in Tanzania [19] and are of serious concern as the heavier the infection, the more severe the morbidity [6]. Therefore, we recommend that all PSAC be included in the target population for MDA with praziquantel to prevent chronic and severe disease [6].

The association between the female sex of PSAC and the prevalence of *S. mansoni* infection could be attributed to the fact that, in Panamasso, girls were more likely to go with their mothers to the pond for domestic activities compared to boys. However, other authors have found no association between sex and the prevalence of *S. mansoni* infection [13, 19, 20]. This may be an indication that boys and girls are equally exposed to infection through water contact [31]. In addition, PSAC who accompanied their parents to the pond were about five times more likely to be infected with *S. mansoni* than those who stayed at home. This corroborates findings of other studies conducted elsewhere [15, 20, 36]. However, in Côte d'Ivoire, Coulibaly et al. found that children who stayed at home were twice more likely to be positive for *S. mansoni* [37]. Altogether, these results indicate that PSAC are at higher risk for *S. mansoni* infection and are a potential reservoir for schistosome parasites, which should benefit from MDA with praziquantel to reach the disease elimination goal [38, 39]. This study showed that PSAC living at low distance from the pond are at threefold increased odds of having *S. mansoni* infection. This is consistent with findings reported in Tanzania [12]. In fact, in Panamasso, the pond is the main source of water for domestic activities for most of the villagers, especially for those living a short distance from the pond despite having well-functioning water supplies. This ensures the continual contamination of the pond with *S. mansoni* eggs due to poor hygiene and sanitation practices at the pond. Moreover, the majority of mothers had no knowledge about means of transmission and of prevention of *S. mansoni* infection. Therefore, implementation of schistosomiasis control measures involving the whole community in this village is needed. These measures should focus on developing participatory health education programs with community members not only to effect continuous behavioral change in parents who expose their young children to *S. mansoni* infection but also to understand the means of disease transmission. Also, involving communities in repairing their drinking water source could help reduce their contact with pond water and disease transmission [31].

We do acknowledge some limitations in this study. First, the results of this study cannot be generalized to other settings. Second, it was not possible to assess the temporal relationship between some of the risk factors and the study outcomes due to the cross-sectional nature of the study. Third, the diarrheic consistency of the stool samples from study participants rendered the preparation of Kato-Katz slides difficult, resulting in underestimation of the prevalence of *S. mansoni* infection [17]*.*

## Conclusion

The present study has shown that *S. mansoni* infection is highly prevalent among PSAC from Panamasso village. The overall GM is of moderate intensity with a high proportion of light infection. Nevertheless, heavy infection was observed in very young children, i.e. those aged 12 to 23 months. Female sex of PSAC, living a short distance from the pond and children’s history of going to the pond were significantly associated with the prevalence of *S. mansoni* infection. The study also revealed that in Panamasso village PSAC’s mothers had no knowledge about means of transmission and prevention of *S. mansoni* infection. These findings stress the need to include all PSAC regardless of their age in the MDA program with praziquantel. However, studies to assess the efficacy and safety of praziquantel in this group of children are warranted. In addition, our findings also call for the development of health education programs on schistosomiasis with community members to effect continuous change in the population’s behavior and to strengthen the provision of safe water.

## Supplementary Information

**Additional file 1: Table S1.** Prevalence of other intestinal parasites according to the parasitological techniques used. The table provided reports the prevalence of intestinal protozoa and intestinal helminths encountered among the study participants using both the Kato-Katz and FEC techniques.

## Data Availability

Data supporting the conclusions of this article are included within the article.

## Abbreviations

AOR: Adjusted odd ratio
CI: Confident interval
COR: Crude odd ratio
epg: Eggs per gram of feces
FEC: Formol ether concentration
GMI: Geometric mean of intensity infection
MDA: Mass drug administration
OR: Odd ratio
POC-CCA: Point-of-care circulating cathodic antigen
PSAC: Preschool-aged children
SAC: School-aged children
SSA: Sub-Saharan Africa

## References

[1] Hotez PJ, Kamath A (2009). Neglected tropical diseases in sub-saharan Africa: review of their prevalence, distribution, and disease burden. PLoS Negl Trop Dis.

[2] van der Werf MJ, de Vlas SJ, Brooker S, Looman CW, Nagelkerke NJ, Habbema JD (2003). Quantification of clinical morbidity associated with schistosome infection in sub-Saharan Africa. Acta Trop.

[3] Mueller A, Fuss A, Ziegler U, Kaatano GM, Mazigo HD (2019). Intestinal schistosomiasis of Ijinga Island, north-western Tanzania: Prevalence, intensity of infection, hepatosplenic morbidities and their associated factors. BMC Infect Dis.

[4] King CH, Dickman K, Tisch DJ (2005). Reassessment of the cost of chronic helmintic infection: a meta-analysis of disability-related outcomes in endemic schistosomiasis. Lancet.

[5] King CH, Dangerfield-Cha M (2008). The unacknowledged impact of chronic schistosomiasis. Chronic Illn.

[6] Nalugwa A, Nuwaha F, Tukahebwa EM, Olsen A (2017). Schistosoma mansoni-associated morbidity among preschool-aged children along the shores of lake Victoria in Uganda. Trop Med Infect Dis.

[7] Stothard JR, Gabrielli AF (2007). Schistosomiasis in African infants and preschool children: to treat or not to treat?. Trends Parasitol.

[8] Hotez PJ, Fenwick A (2009). Schistosomiasis in Africa: an emerging tragedy in our new global health decade. PLoS Negl Trop Dis.

[9] Mutapi F (2015). Changing policy and practice in the control of pediatric schistosomiasis. Pediatrics.

[10] Odogwu SE, Ramamurthy NK, Kabatereine NB, Kazibwe F, Tukahebwa E, Webster JP (2006). Schistosoma mansoni in infants (aged < 3 years) along the Ugandan shoreline of Lake Victoria. Ann Trop Med Parasitol.

[11] Sacolo-gwebu H, Chimbari M, Kalinda C (2019). Prevalence and risk factors of schistosomiasis and soil-transmitted helminthiases among preschool aged children (1–5 years) in rural KwaZulu-Natal, South Africa: a cross-sectional study. Infect Dis Poverty.

[12] Okoyo C, Simiyu E, Njenga SM, Mwandawiro C (2018). Comparing the performance of circulating cathodic antigen and Kato-Katz techniques in evaluating Schistosoma mansoni infection in areas with low prevalence in selected counties of Kenya : a cross- sectional study. BMC Public Health.

[13] Alemu A, Tegegne Y, Damte D, Melku M (2016). Schistosoma mansoni and soil-transmitted helminths among preschool-aged children in Chuahit, Dembia district, Northwest Ethiopia: prevalence, intensity of infection and associated risk factors. BMC Public Health.

[14] Goran KN (2012). Efficacy and safety of praziquantel in preschool-aged children in an area co-endemic for *Schistosoma mansoni* and *S. haematobium*. PLoS Negl Trop Dis..

[15] Kemal M, Tadesse G, Esmael A, Abay SM, Kebede T (2019). Schistosoma mansoni infection among preschool age children attending Erer Health Center, Ethiopia and the response rate to praziquantel. BMC Res Notes.

[16] Armoo S, Cunningham LJ, Campbell SJ, Aboagye FT, Boampong FK, Hamidu BA (2020). Detecting Schistosoma mansoni infections among pre-school-aged children in southern Ghana: a diagnostic comparison of urine-CCA, real-time PCR and Kato-Katz assays. BMC Infect Dis.

[17] Coulibaly JT, N’Gbesso YK, Knopp S, N’Guessan NA, Silué KD, van Dam GJ (2013). Accuracy of urine circulating cathodic antigen test for the diagnosis of Schistosoma mansoni in preschool-aged children before and after treatment. PLoS Negl Trop Dis.

[18] Garba A, Barkiré N, Djibo A, Lamine MS, Sofo B, Gouvras AN (2010). Schistosomiasis in infants and preschool-aged children: Infection in a single Schistosoma haematobium and a mixed *S. haematobium*–*S. mansoni* foci of Niger. Acta Trop..

[19] Ruganuza DM, Mazigo HD, Waihenya R, Morona D, Mkoji GM (2015). Schistosoma mansoni among pre-school children in Musozi village, Ukerewe Island, North-Western-Tanzania: prevalence and associated risk factors. Parasit Vectors.

[20] Nalugwa A, Olsen A, Tukahebwa ME, Nuwaha F (2015). Intestinal schistosomiasis among preschool children along the shores of Lake Victoria in Uganda. Acta Trop.

[21] Poda JN, Sawadogo L (1994). Hôtes intermédiaires et prévalence de la bilharziose au Burkina Faso. Sci Tech.

[22] Gabrielli AF, Toure S, Sellin B, Sellin E, Ky C, Ouedraogo H (2006). A combined school- and community-based campaign targeting all school-age children of Burkina Faso against schistosomiasis and soil- transmitted helminthiasis: performance, financial costs and implications for sustainability. Acta Trop.

[23] Zongo D, Kabre BG, Dayeri D (2013). Profil parasitologique de deux formes de schistosomiase (formes urinaire et intestinale) dans dix sites du Burkina Faso. CR Biol.

[24] Bagayan M, Zongo D, Oueda A, Sorgho H, Savadogo B, Drabo F (2016). Prévalence de la schistosomose et des géohelminthiases chez des élèves de l’école primaire au Burkina Faso. Med Sante Trop.

[25] Dianou D, Poda JN, Sawadogo LG, Sorgho H, Wango SP, Sondo B (2004). Parasitoses intestinales dans la zone du complexe hydroagricole du Sourou au Burkina Faso. VertigO-La Rev en Sci l’environnement.

[26] Ganaba KSM. Étude de la biodiversité végétale urbaine de l’Afrique de l’Ouest. Cas de la ville de Bobo-Dioulasso. Ecole nationale des eaux et forêts de Dinderesso, Rapport de Stage; 2017.

[27] Katz N, Chaves A, Pellegrino J (1972). A simple device for quantitative stool thick-smear technique in *Schistosomiasis mansoni*. Rev Inst Med Trop Sao Paulo.

[28] WHO. Guidelines for the evaluation of soil-transmitted helminthiasis and schistosomiasis at community level. A guide for managers of control programmes. 1998. http://whqlibdoc.who.int/hq/1998/WHO_CTD_SIP_98.1.pdf. Accessed 30 Aug 2009

[29] Ritchie LS (1948). An ether sedimentation technique for routine stool examinations. Bull US Army Med Dep.

[30] Peralta M, Cavalcanti MG (2018). Is POC-CCA a truly reliable test for schistosomiasis diagnosis in low endemic areas? The trace results controversy. PLoS Negl Trop Dis.

[31] Dabo A, Badawi HM, Bary B, Doumbo OK (2011). Urinary schistosomiasis among preschool-aged children in Sahelian rural communities in Mali. Parasit Vectors.

[32] Sousa-figueiredo C, Betson M, Kabatereine NB, Stothard JR (2013). The urine circulating cathodic antigen (CCA) dipstick: a valid substitute for microscopy for mapping and point-of-care diagnosis of intestinal schistosomiasis. PLoS Negl Trop Dis.

[33] Kittur N, Castleman JD, CHC, King CH, Colley DG. (2016). Comparison of schistosoma mansoni prevalence and intensity of infection, as determined by the circulating cathodic antigen urine assay or by the Kato-Katz Fecal assay: a systematic review. Am J Trop Med Hyg.

[34] Ekpo UF, Oluwole AS, Abe EM, Etta HE, Olamiju F, Mafiana CF (2012). Schistosomiasis in infants and pre-school-aged children in sub-Saharan Africa: implication for control. Parasitol.

[35] ANOFEL. Parasitoses et mycoses des régions tempérées et tropicales, 6 ed. Paris: Elsevier; 2019. 552 p.

[36] Sousa-figueiredo JC, Pleasant J, Day M, Betson M, Rollinson D, Montresor A (2010). Treatment of intestinal schistosomiasis in Ugandan preschool children: best diagnosis, treatment efficacy and side-effects, and an extended praziquantel dosing pole. Int Heal.

[37] Coulibaly JT, Gbesso YKN, Guessan NAN, Winkler MS (2013). Epidemiology of schistosomiasis in two high-risk communities of south Côte d’Ivoire with particular emphasis on pre-school-aged children. Am J Trop Med Hyg.

[38] WHO. World Health Assembly resolution. 2012. WHA65.21 http://www.who.int/neglected_diseases/Schistosomiasis_wha65/en. Accessed 2 June 2016.

[39] Zwang J, Olliaro P (2017). Efficacy and safety of praziquantel 40 mg/kg in preschool-aged and school-aged children: a meta-analysis. Parasit Vectors.

