# Supplementary material for: Prevalence and risk factors of Schistosoma mansoni infection among preschool-aged children from Panamasso village, Burkina Faso
Source: Parasit Vectors. 2021 Apr 1;14:185. doi: 10.1186/s13071-021-04692-8 (PMC8017716; doi:10.1186/s13071-021-04692-8)
Supplement: Supplementary file 1 — Additional file 1: Table S1. Prevalence of other intestinal parasites according to the parasitological techniques used. The table provided reports the prevalence of intestinal protozoa and intestinal helminths encountered among the study participants using both the Kato-Katz and FEC techniques. [file 13071_2021_4692_MOESM1_ESM.docx]

**Table S1 : Prevalence of other intestinal parasites according to the parasitological techniques used**

| Parasites | Parasitological techniques | |
| --- | --- | --- |
|  | **Kato Katz (n, %)** | **FEC (n, %)** |
| Protozoa | 0 (0.0) | 92 (40.4) |
| *Entamoeba coli* | - | 48 (21.0) |
| *Giardia intestinalis* | - | 27 (11.8) |
| *Entamoeba histolytica minuta* | - | 23 (10.0) |
| *Endolimax nana* | - | 8 (3.5) |
| *Isospora belli* | - | 1 (0.4) |
| Helminths | 6 (2.6) | 7 (3.1) |
| *Enterobius vermicularis* | 4 (1.8) | 2 (0.9) |
| *Trichuris trichiura* | 1 (0.4) | 1 (0.4) |
| *Dicrocoelium dendriticum* | 1 (0.4) | 0 (0.0) |
| *Hymenolepis nana* | 0 (0.0) | 3 (1.3) |
| *Necator americanus* | 0 (0.0) | 1 (0.4) |
